# Association of the dose of maternal general anaesthesia during Caesarean delivery with 5-minute Apgar scores: a retrospective single centre cohort study

**DOI:** 10.1016/j.bjao.2026.100534

**Published:** 2026-02-27

**Authors:** Shubhangi Singh, Thomas T. Klumpner, Baskar Rajala, Jefferine Li, Monica Servin, Robert Schumacher, Alissa Carver, Carlo Pancaro, Nicole Barrios, Yuan Yuan, Graciela Mentz, Joanna A. Kountanis

**Affiliations:** 1 Department of Anesthesiology, University of Michigan, Ann Arbor, MI, USA; 2 Department of Obstetrics & Gynecology, University of Michigan, Ann Arbor, MI, USA; 3 Department of Anesthesiology, University of California, Irvine, CA, USA; 4 Ambulatory Anesthesia Solutions, Novi, MI, USA; 5 Department of Pediatrics, University of Michigan, Ann Arbor, MI, USA; 6 Wilmington Maternal-Fetal Medicine, Wilmington, NC, USA

**Keywords:** Apgar scores, Caesarean delivery, Caesarean hysterectomy, general anaesthesia, maternal general anaesthetic, neonatal outcomes

## Abstract

**Background:**

Neuraxial anaesthesia is preferred for Caesarean deliveries, but general anaesthesia is used in situations such as emergencies, contraindications to neuraxial anaesthesia, or anticipated complex deliveries. Although previous research has examined how the *duration* of fetal general anaesthesia exposure affects neonatal outcomes, the *total dose* of general anaesthesia has not been studied**.** The primary aim was to determine the association, if any, between the general anaesthesia dose and 5-min neonatal Apgar scores. We hypothesised that greater maternal exposure to general anaesthesia would be associated with lower neonatal 5-min Apgar scores.

**Methods:**

This retrospective single centre cohort study evaluated the association between total maternal general anaesthesia dose and 5-min Apgar scores among neonates born *via* planned Caesarean delivery under general anaesthesia between January 2013 and March 2022. Total general anaesthesia dose was quantified using the area under the curve of effective minimum alveolar concentration (EMAC-AUC) from general anaesthesia induction to neonatal delivery (T). Multivariable logistic regression adjusted for maternal age, gestational age, and maternal hypertension. Maternal blood pressure was quantified by the area under the curve for mean arterial pressure <80 mm Hg (MAP80AUC).

**Results:**

Analysis included 101 neonates born via planned Caesarean delivery under maternal general anaesthesia. Every 5-MAC-minute increase in EMAC-AUC was associated with a 10% increase in the adjusted odds of a 5-min Apgar score of <7 (odds ratio, 1.10; 95% confidence interval, 1.05–1.22; *P* <0.001). However, this association was not significant after adjusting for maternal MAP80AUC (odds ratio, 1.05; 95% confidence interval, 1.00–1.16; *P*=0.177).

**Conclusions:**

In this cohort of preterm, late preterm, and term neonates delivered *via* planned CD or Caesarean hysterectomy, total general anaesthetic dose was statistically associated with the 5-min Apgar score, although the clinical effect was modest. The attenuation of the EMAC-AUC association after adjusting for MAP80AUC suggests that maternal hypotension may play an important explanatory role, although causality cannot be inferred. Maintaining mean arterial pressure >80 mm Hg may reduce the likelihood of low Apgar scores, although prospective studies are needed to confirm this relationship.

Neuraxial anaesthesia is the preferred type of anaesthesia for Caesarean deliveries (CDs) in obstetric practice, given worse maternal and neonatal outcomes demonstrated with general anaesthesia.^1^^,^^2^ Therefore, general anaesthesia is generally reserved for settings such as emergencies,^3^ maternal refusal of neuraxial anaesthesia, when contraindications to neuraxial anaesthesia exist, or in settings of a failed neuraxial anaesthesia. General anaesthesia may also be preferred when massive postpartum haemorrhage (PPH) is anticipated, such as during Caesarean hysterectomies as a result of placenta accreta spectrum (PAS) disorders.^4^^,^^5^

Clinical evidence suggests that general anaesthesia is associated with worse neonatal outcomes compared with neuraxial anaesthesia for CD,^1^^,^^2^ especially for an already acutely compromised fetus.^6^^,^^7^ These associations, however, may be confounded by the clinical scenarios necessitating general anaesthesia, which often involve fetal compromise, although the role of the transfer of anaesthetics *via* the placenta^8^ cannot be ruled out. Most research comparing maternal general anaesthesia with neuraxial anaesthesia focuses on the influence of the time interval between the beginning of the anaesthetic and the delivery of the fetus.^9^^,^^10^ However, to our knowledge, no studies in the literature have yet evaluated the effect of the total dose of maternal general anaesthetic agents on neonatal outcomes when the fetus is not acutely compromised. We therefore designed this study to better understand the association between the total dose of general anaesthesia and 5-min Apgar scores among neonates born *via* planned CDs and Caesarean hysterectomies. The primary aim was to determine the association, if any, between the general anaesthesia dose and 5-min neonatal Apgar scores. We hypothesised that greater maternal exposure to general anaesthesia would be associated with lower neonatal 5-min Apgar scores.

## Methods

This study was approved by the Institutional Review Board at Michigan Medicine (IRB number: HUM00203655; date of approval: 10 September 2021, last amended: 1 July 2024). This was a single-centre retrospective cohort study of neonates born *via* planned CD or Caesarean hysterectomies under general anaesthesia from January 2013 to 14 March 2022. This manuscript follows the Strengthening the Reporting of Observational Studies in Epidemiology (STROBE) guidelines for observational cohort studies.

### Study cohort

#### Inclusion criteria

Any singleton neonate born *via* planned CD or Caesarean hysterectomy where general anaesthesia was used before the delivery between January 2013 and 14 March 2022 was included. In this study, a planned CD or Caesarean hysterectomy denotes a procedure that was scheduled antenatally and performed without any urgent indications and without a setting of emergency. To obtain the study cohort, we identified all CDs and Caesarean hysterectomies done under general anaesthesia. Because a single query did not reliably identify *planned/scheduled* cases, a two-pronged approach was used: (1) Multicenter Perioperative Outcomes Group (MPOG) Quality measure GA-01-OB was used. Founded in 2008, MPOG is an international consortium of institutions that share anaesthetic data *via* a common data model for research, education, and quality improvement (QI).^11^ Participating sites’ data are available on their local MPOG server for single-centre research. This quality measure (GA-01-OB) was designed by a committee of experts in obstetric anaesthesiology to determine the percentage of CDs performed under general anaesthesia.^12^ A detailed description of the MPOG methodology can be found elsewhere.^13^ Because the GA-01-OB measure excludes patients who have a diagnosis of PAS or PPH (per International Classification of Diseases [ICD]-9 and -10 codes), this search was expanded to include patients with an MPOG phenotype of ‘general anaesthesia’ and ICD-9 or ICD-10 codes for PPH, retained placenta (ICD-9), and PAS (ICD-10) (Supplementary Table 1) to find cases of Caesarean hysterectomy done for PAS under general anaesthesia. (2) A QI database of all Caesarean hysterectomies performed at Michigan Medicine, that has been kept by the Obstetrics and Gynaecology department since 2013, was searched to identify neoantes born via Caesarean hysterectomies performed under general anaesthesia that may not have been detected by the MPOG queries.

#### Exclusion criteria

Neonates born *via* emergency CD (identified by ASA PS ‘E’, a delivery indication of non-reassuring fetal status or fetal bradycardia) were excluded. Intrapartum Caesarean hysterectomies, intrapartum CDs, and *ex utero* intrapartum (EXIT) procedures were also excluded. Patients with multiple gestation were excluded. Data regarding congenital fetal anomalies were extracted electronically (Supplementary Table 1) and the cases flagged and reviewed manually. Neonates with an antepartum diagnosis of major pulmonary (congenital diaphragmatic hernia, congenital pulmonary airway malformation) or major cardiac anomalies (tetralogy of Fallot, transposition of great vessels, hypoplastic left heart syndrome, total anomalous pulmonary venous connection) were also excluded. Procedures in which ketamine or etomidate were administered as induction agents were excluded as they are rarely used and are more likely to be associated with an emergency CD. Further, any cases where the first bolus of propofol was given after the delivery of the neonate were excluded. The medical records of all the remaining cases were manually reviewed to exclude those who had a non-reassuring fetal status, an intrapartum CD, any major pulmonary cardiac anomalies as above, and where the information obtained from the electronic data pull may not have been enough to identify these exclusions. This was done to ensure strict adherence to the exclusion criteria.

### Primary exposure of interest

The primary exposure of interest was the total dose of the general anaesthesia administered. Most commonly, inhalation anaesthetics (sevoflurane and isoflurane) are used to maintain anaesthesia before baby delivery at our institution. The minimum alveolar concentration (MAC) is a standard measure of potency of inhaled anaesthetic agents and is defined as the concentration at which 50% of people do not move in response to a painful stimulus.^14^ We electronically extracted minute-to-minute end-tidal concentration of the inhalation anaesthetic and then calculated the MAC value. For propofol, the plasma concentrations at which 50% of patients do not move in response to a noxious stimulus (also called Cp50) has been used as the analogous concept of 1 MAC.^15^ Because we do not have the technology to measure it at our institution, we used 150 μg kg^−1^ min^−1^ as the equivalent to 1 MAC for propofol as quoted by previous work.^16^ If dexmedetomidine was administered at ≥0.2 μg kg^−1^ h^−1^, the MAC was multiplied by 2.^17^ Thus, the following equation was used for calculating an ‘effective MAC’.^16^

Effective MAC=End-tidal Sevoflurane %/2+End-tidal Isoflurane %/1.2+End-tidal Desflurane %/6+End-tidal Nitrous %/105+Propofol (10 mg/1 ml) Infusion/150.

The general anaesthesia dose was quantified in terms of effective MAC-time units. One MAC-hour, defined as exposure to 1 MAC of an inhalation anaesthetic for 1 h, is a well-known concept and has been used to quantify anaesthetic dose in previous studies.^18^ This was calculated using the effective-MAC value and the duration of fetal exposure to general anaesthesia in the form of area under the curve (AUC), referred to as EMAC-AUC in this manuscript. We described this in units of MAC-minutes because we anticipated that the duration of exposure among some patients would be very short. The duration of fetal exposure to general anaesthesia (T) was the time from induction of anaesthesia (time of first propofol administration) to the delivery of the neonate. Although it is generally known that MAC requirement decreases in pregnancy, the exact pregnancy-specific algorithms are not available. Therefore, we were unable to adjust for that. Because propofol boluses and opioids are not accounted for by the above equation, they were treated as confounders as described below.

The following confounding factors were also collected from each anaesthetic record: propofol bolus dose (total amount including the induction dose and any other boluses given) before delivery of the baby, benzodiazepine administration (binary), total dose of ultra-short-acting opioid remifentanil (total amount), administration of opioid other than remifentanil (binary), maternal hypoxia (SpO_2_ <90% for ≥5 minutes) (binary), maternal hypercarbia (end-tidal carbon dioxide [ETCO_2_] >45 mm Hg for ≥5 min) (binary), and maternal blood pressure. Maternal blood pressure was quantified by calculating the AUC (duration × magnitude) for systolic blood pressure (SBP) <100 mm Hg (SBP100AUC)^19^, SBP <90 mm Hg (SBP90AUC)^20^, mean arterial pressure (MAP) <80 mm Hg (MAP80AUC),^19^ and MAP <65 mm Hg (MAP65AUC)^20^ during time T. In the presence of an arterial line, the invasive blood pressure (which gives blood pressure measurement with every beat) was used for calculation of the maternal blood pressure, and noninvasive blood pressure (measured every 1–3 min) was used in its absence.

Maternal age and BMI, neonatal gestational age, gravidity and parity at delivery, steroid administration before delivery, uterine incision to delivery time, a diagnosis of hypertension (HTN), and diabetes mellitus (chronic and gestational) during the pregnancy episode were electronically collected from the maternal electronic medical record (EMR) (Supplementary Table 1). HTN was a composite of chronic HTN, gestational HTN, and pre-eclampsia with or without severe features or a combination of these diagnoses. A patient was defined as steroid complete if they had completed at least one course of antenatal betamethasone (consisting of two doses of 12 mg i.m., 24 h. apart) or dexamethasone (four doses of 6 mg, 12 h apart).^21^

The primary outcome of neonatal 5-min Apgar scores was electronically extracted from the neonatal EMR. All CDs done under general anaesthesia are attended by a paediatric team if ≥36 weeks of gestation and by a neonatology team if <36 weeks. The paediatric and the neonatology teams assign the Apgar scores. We chose the cut-off value of 7 for neonatal 5-min Apgar scores because a 5-min Apgar score <7 has been associated with neonatal mortality, morbidity, and poor long-term neurodevelopmental outcomes.^22^ This measure has also been used in many previous studies.^9^^,^^10^^,^^19^ Secondary outcomes included neonatal 1-min Apgar score, umbilical cord blood arterial and venous pH <7.1,^23^^,^^24^ and the presence of transient tachypnoea of newborn (TTN), all obtained from the neonatal EMR (Supplementary Table 1). A base deficit of ≥12 mmol L^−1^ was also collected as it is a well-established indicator of neonatal compromise^19^^,^^24^^,^^25^ and has been associated with long-term neurodevelopmental disability^26^ and an intelligence quotient <70. The electronic record was also manually reviewed for additional secondary outcomes, including the requirement for continuous positive airway pressure (CPAP) for ≥2 h, supplemental oxygen (FiO_2_ ≥0.3) for ≥4 h, need and duration of neonatal intubation, and admission to the neonatal intensive care unit (NICU).^9^

The manual data collection was done by the authors SS, BR, and JL. A standard template was created for data collection by author consensus. In cases of disagreement, input was sought from the senior authors: TTK, CP, and JAK. The decision of the senior author JAK was final.

### Statistical analysis

Power analysis and sample size determination were done following the approach recommended by Li and colleagues.^27^ Assumptions for this analysis were alpha 0.05, 95% power, and a conservative 0.5 intraclass correlation to accommodate potential clustering. In addition, we assumed a potential 10% data drop-off. A final sample size estimate of 80–100 patients was expected to provide 95% power to test our research question. Because the QI database was initiated in 2013, we selected that date as the beginning of data collection. The final data were extracted in June 2022; therefore, to ensure availability of reliable data, we selected the endpoint to be 14 March 2022. This gave us 101 final cases after applying our strict exclusion criteria, thus providing sufficient power to address our research question. The data collection was therefore not extended beyond this period.

The distributions of primary and secondary outcomes were described as means and standard deviations, medians, and interquartile range (IQR), or frequencies as a part of the initial exploratory data analysis (EDA) and this was used to inform the final modelling strategy. Histograms and QQ-plots were used for continuous outcomes. Extreme values were identified using the Tukey–Fences approach and their potential removal from the analysis was determined. Missing values, patterns, and rates were examined. Missing rates were <5% so complete case analysis is reported here.

To assess the association between the EMAC-AUC and neonatal 5-min Apgar scores, we used the generalised estimating equations (GEE) approach, with logit link for the outcome, which was binary (i.e. <7 and ≥7). The GEE approach does not require explicit identification of the clustering process; rather, the approach to clustering adjustments is implicit in the working correlation matrix used (e.g. exchangeable). The multivariable model was built to assess the relationship between EMAC-AUC (primary exposure variable) and neonatal 5-min Apgar score (primary outcome) after adjusting for confounding exposure variables. Exposure variables that were found significant in the univariable analysis with the best goodness of fit were considered for this multivariable model based on the authors’ clinical judgement of their effect on the 5-min Apgar scores (further described below). Goodness of fit was assessed using Akaike information criterion (AIC) and Bayesian information criterion (BIC). Pearson’s correlation and variance inflation factor (VIF) were used to assess multicollinearity of confounders and predictors considered for the multivariable models. Final variables included were those that did not exhibit collinearity as measured by VIF <5.^28^^,^^29^

#### Selection of variables for the multivariable model

Gestational age at delivery and steroid completion were found to be correlated, with a correlation coefficient of −0.76. This is expected clinically, as only the mothers of neonates expected to be born preterm would receive steroids. Therefore, we elected to not include steroid completion in the final model. MAP has been used as a marker of hypotension in previous studies^19^^,^^30^^,^^31^ and correlates with neonatal outcomes,^19^^,^^31^ reflecting the dependence of organ and placental perfusion on MAP. Also, the univariable analysis showed MAP80AUC to be significantly correlated to 5-min Apgar with the best goodness of fit of all the blood pressure parameters. Consequently, we elected to include MAP80AUC as the blood pressure parameter in the final model. We did not include parity in the final model as it does not have a direct clinical effect on the 5-min Apgar scores. Patient age was included as the demographic variable. Hence, the variables that we adjusted for in the final models included maternal age, gestational age, and MAP80AUC. HTN was included *post hoc* in the model to test for interaction, if any, between HTN and MAP80AUC. SAS version 9.4 (SAS Institute Inc. Cary, North Carolina, USA) was used for all analyses. We evaluated the classification performance of the multivariable model using the area under the receiver operating curve (AUC-ROC) statistic, with a high (>0.7) value as the threshold to indicate an acceptable model classification performance.

## Results

**Fig 1:**
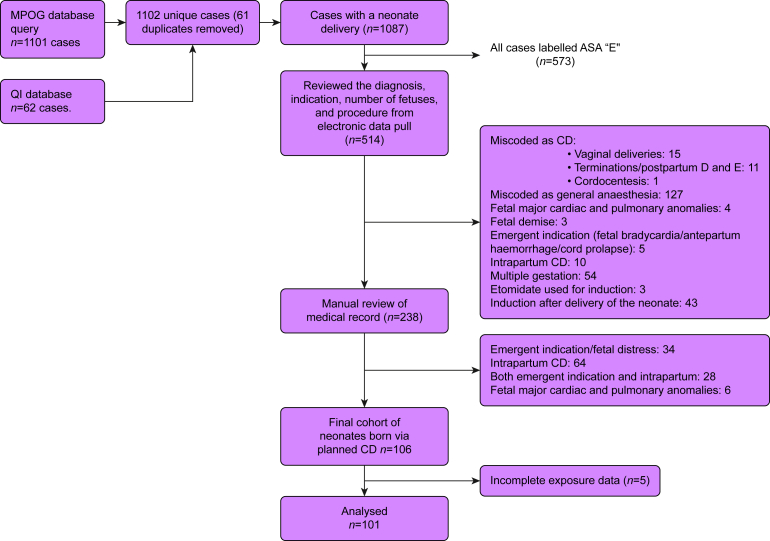
Constitution of the study cohort consisting of planned Caesarean deliveries and Caesarean hysterectomies under general anaesthesia. CD, Caesarean delivery; MPOG, Multicenter Perioperative outcomes group; QI, quality improvement.

A total of 1101 cases were identified using the MPOG queries described above, and 62 surgical cases were identified from the QI database. After removing duplicates, 1102 unique surgical cases were found, of which 1087 had a birth via Caesarean delivery associated with them. Information derived as part of the electronic data pull such as case booking ‘diagnosis’, and ‘procedure’ was reviewed, leading to exclusion of 849 cases. Manual review of the EMR was completed for the remaining 238 neonates and their mothers, further revealing an additional 132 cases that met exclusion criteria. Finally, five cases were excluded because of incomplete maternal exposure data in the EMR, ultimately yielding a study cohort of 101 neonates (Fig. 1). In the final cohort, there were 41 cases of planned neonatal births via Caesarean hysterectomies and 60 cases of neonatal births via planned CDs with general anaesthesia. The indications for CD and reasons for the use of general anaesthesia are shown in Table 1.

**Table 1:** Baseline and exposure characteristics of the cohort (*N*=101). Continuous data (maternal age, BMI, uterine incision to delivery time, gestational age, EMAC-AUC, total remifentanil dose before delivery, propofol bolus dose before delivery, SBP100AUC, SBP90AUC, MAP80AUC, and MAP65AUC) are described as mean (sd), ordinal data (gravidity, parity) and non-normally distributed continuous data (time from induction to baby delivery) are described as median (interquartile range), and categorical data (gestational age of neonates, steroid completion, HTN, diabetes, primary indication for, CD and primary reason for general anaesthesia) are describes as *n* (%). CD, Caesarean delivery; EMAC-AUC, effective minimum alveolar concentration area under the curve; HTN, hypertension; MAP80AUC, mean arterial pressure <80 mm Hg area under the curve described as mm Hg-minutes; MAP65AUC, mean arterial pressure <65 mm Hg area under the curve described as mm Hg-minutes; PAS, placenta accreta spectrum; SBP100AUC, systolic blood pressure <100 mm Hg area under the curve described as mm Hg-minutes; SBP90AUC, systolic blood pressure <90 mm Hg area under the curve described as mm Hg-minutes. ∗The variable HTN was a composite of chronic hypertension, gestational hypertension, and pre-eclampsia with and without severe features. ^†^Includes all opioids except remifentanil. Fentanyl, morphine, and hydromorphone were the only other opioids given in this cohort.

	Full sample	5-min Apgar score <7	5-min Apgar score ≥7
	(*n*=101)	(*n*=36)	(*n*=65)
Baseline characteristics			
Maternal age (yr)	31.9 (5.6)	32.6 (5.9)	31.6 (5.4)
BMI (kg m^−2^)	33.9 (8.1)	34.8 (8.8)	33.4 (7.8)
Uterine incision to delivery (min)	1.9 (2.7)	1.9 (1.1)	1.9 (3.3)
Gestational age (weeks)	36.2 (2.5)	35.3 (2.2)	36.7 (2.5)
Gestational age of neonates			
Extreme preterm (<28 weeks)	0 (0)	0 (0)	0 (0)
Very preterm (28–<32 weeks)	4 (4.0)	2 (5.6)	2 (3.1)
Moderate preterm (32–<34 weeks)	11 (10.9)	5 (13.9)	6 (9.2)
Late preterm (34–<37 weeks)	43 (42.6)	19 (52.8)	24 (36.9)
Term (37 weeks and higher)	43 (42.6)	10 (27.8)	33 (50.8)
Gravidity	4 (2–5)	4 (2–5)	3 (2–5)
Parity	2 (1–3)	2 (1–3)	1 (1–2)
Steroid completion	59 (58.4)	28 (77.8)	31 (47.7)
HTN∗	29 (28.7)	12 (33.3)	17 (26.2)
Chronic hypertension	12 (11.9)	4 (11.1)	8 (12.3)
Gestational hypertension	19 (18.8)	8 (22.2)	11 (16.9)
Pre-eclampsia without severe features	0 (0)	0 (0)	0 (0)
Pre-eclampsia with severe features	6 (5.9)	2 (5.6)	4 (6.2)
Diabetes	18 (17.8)	8 (22.2)	10 (15.4)
Primary indication for CD			
Suspected PAS	46 (45.5)	25 (69.4)	21 (32.3)
Repeat CD	27 (26.7)	6 (16.7)	21 (32.3)
Contraindications to vaginal delivery	16 (15.8)	3 (8.3)	13 (20.0)
Breech or other non-cephalic presentation	6 (5.9)	2 (5.6)	4 (6.2)
Maternal request	6 (5.9)	0 (0)	6 (9.2)
Primary reason for general anaesthesia			
Anticipated high blood loss	47 (46.5)	25 (69.4)	22 (33.8)
Contraindications to neuraxial anaesthesia	27 (26.7)	6 (16.7)	21 (32.3)
Failed neuraxial anaesthesia	16 (15.8)	3 (8.3)	13 (20.0)
Patient refusal to neuraxial anaesthesia	11 (10.9)	2 (5.6)	9 (13.8)
Intraoperative characteristics			
Time from induction to baby delivery (T) (min)	19 (7 to 67)	64 (19.5 to 81)	11 (5 to 44)
EMAC-AUC (MAC-min)	29.7 (34.3)	47.4 (36.3)	19.9 (29.1)
Total remifentanil dose before delivery (mcg)	64.0 (384.6)	50.3 (175.4)	71.5 (462.8)
Propofol bolus dose before delivery (mg)	203.6 (56.4)	202.4 (69.4)	204.3 (48.3)
Midazolam administration	29 (28.7)	13 (36.1)	16 (24.6)
Opioid administration^†^	42 (41.6)	13 (36.1)	29 (44.6)
SBP100AUC (mm Hg-min)	41.2 (87.5)	67.1 (117.2)	26.88 (62.2)
SBP90AUC (mm Hg-min)	11.5 (26.8)	16.4 (29.9)	8.8 (24.7)
MAP80AUC (mm Hg-min)	137.5 (220.6)	254.1 (285.7)	72.9 (139.6)
MAP65AUC (mm Hg-min)	16.6 (37.1)	25.9 (41.9)	11.5 (33.4)

The patient and obstetric characteristics of our study cohort are summarised in Table 1.

The variables that were found to have a statistical association with 5-min Apgar score <7 in the univariable analysis were EMAC-AUC, SBP100AUC, MAP80AUC, MAP65AUC, gestational age at delivery, steroid completion, and parity (Table 2).

**Table 2:** Univariable generalised estimating equations model showing the association of primary and secondary exposure parameters on 5-min Apgar score <7. CI, confidence interval; EMAC-AUC, effective minimum alveolar concentration area under the curve; HTN, hypertension; MAP80AUC, mean arterial pressure <80 mm Hg area under the curve; MAP65AUC, mean arterial pressure <65 mm Hg area under the curve; OR, odds ratio; SBP100AUC, systolic blood pressure <100 mm Hg area under the curve; SBP90AUC, systolic blood pressure <90 mm Hg area under the curve. ∗Odds ratio for each 5-unit increment. ^†^The variable HTN was a composite of chronic hypertension, gestational hypertension, and pre-eclampsia with and without severe features.

Parameter	OR (95% CI)	*P*-value
Maternal age	1.04 (0.97–1.10)	0.255
BMI	1.01 (0.97–1.06)	0.570
EMAC-AUC∗	1.16 (1.05–1.22)	<0.001∗
SBP100AUC∗	1.00 (1.00–1.05)	0.015∗
SBP90AUC∗	1.05 (1.00–1.10)	0.080
MAP80AUC∗	1.00 (1.00–1.05)	<0.001∗
MAP65AUC∗	1.05 (1.00–1.10)	0.022∗
Uterine incision to delivery time	1.00 (0.89–1.13)	0.965
Gestational age at delivery	0.79 (0.68–0.92)	0.002∗
Total remifentanil dose before delivery	1.00 (1.00–1.00)	0.613
Propofol bolus dose before delivery	0.99 (0.99–1.00)	0.083
Gravidity	1.04 (0.89–1.21)	0.603
Parity	1.30 (1.03–1.63)	0.024∗
Steroid complete	3.74 (1.71–8.18)	0.001∗
Midazolam administration	1.72 (0.85–3.52)	0.134
Opioid administration	0.66 (0.34–1.30)	0.231
Abruption	1.90 (0.38–9.44)	0.431
HTN^†^	1.29 (0.60–2.75)	0.510
Diabetes	1.36 (0.59–3.11)	0.472

**Fig 2:**
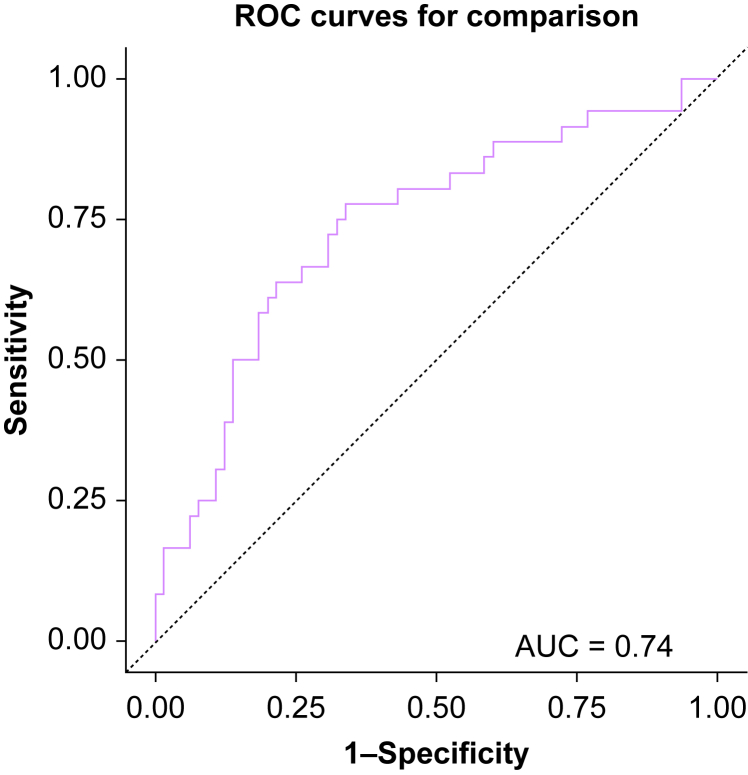
ROC (Solid purple) for the multivariable model (EMAC-AUC, maternal age, gestational age at delivery, and HTN) for predicting 5-min Apgar scores. The ROC shows an AUC of 0.74 (95% confidence interval, 0.64–0.84). AUC, area under the curve; EMAC-AUC, effective minimum alveolar concentration area under the curve; HTN, hypertension (composite of chronic hypertension, gestational hypertension, pre-eclampsia with and without severe features); ROC, receiver operating curve.

The AUC-ROC for the multivariable model including EMAC-AUC, maternal age, gestational age at delivery, and HTN for predicting a 5-min Apgar score <7 was 0.74 (95% confidence interval, 0.64–0.84), suggesting that this model is performing better than a random guess (Fig. 2). EMAC-AUC had a statistically significant association with the 5-min Apgar score, with every 5-MAC-min increase in EMAC-AUC being associated with a clinically small, 10%, increase in adjusted odds of 5-min Apgar score <7. Adjustment for MAP80AUC completely attenuated the association between EMAC-AUC and 5-min Apgar scores (Table 3). No interaction could be demonstrated between MAP80AUC and HTN.

**Table 3:** Association between the EMAC-AUC and 5-min Apgar score <7 when adjusted for confounding variables. AIC, Akaike information criterion; BIC, Bayesian information criterion; EMAC-AUC, effective minimal alveolar concentration area under the curve over time; HTN, hypertension (including chronic hypertension, gestational hypertension, and pre-eclampsia with or without severe features); MAP80AUC, area under the curve for mean arterial pressure <80 mm Hg over time. ∗Odds ratio for each 5-unit increment. ^†^Primary exposure: EMAC-AUC; primary outcome: 5-min Apgar score. ^‡^HTN was added *post hoc* in order to evaluate the interaction between MAP80AUC and HTN, as no interaction was found, the interaction variable was removed for the final model.

Exposure and outcome	Odds ratio∗	*P*-value	Goodness of fit		Model adjusted for
			AIC	BIC	
EMAC-AUC→5-min Apgar score <7^†^	1.10 (1.05–1.22)	<0.001	188.13	201.20	Maternal age, gestational age at delivery, HTN^‡^
EMAC-AUC→5-min Apgar score <7^†^	1.05 (1.00–1.16)	0.177	183.53	199.22	Maternal age, gestational age at delivery, HTN, and MAP80AUC^‡^

The other neonatal outcomes are described in detail in Supplementary Table 2.

## Discussion

In this cohort of preterm, late preterm, and term neonates delivered *via* planned CD or Caesarean hysterectomy, total general anaesthesia dose was statistically associated with the 5-min Apgar score, although the clinical effect was modest (a 10% increase in odds of 5-min Apgar <7 per 5-MAC-min increase in general anaesthesia exposure). Adjustment for MAP80AUC attenuated the association between EMAC-AUC and Apgar scores. This suggests that maternal hypotension may play an important explanatory role, although causality cannot be inferred. This effect remained consistent among patients with chronic or gestational HTN, with or without pre-eclampsia, as no interaction was observed between HTN and MAP80AUC, suggesting that this finding can be applied to patients with hypertensive disorders.

The effect of general anaesthesia on neonatal outcomes has been studied widely in the literature,^9^^,^^10^^,^^32^^,^^33^ but our study is the first to evaluate the relationship between the dose of general anaesthesia and neonatal outcomes. A recent study demonstrated that in patients with reassuring fetal status, neonatal outcomes were similar between neuraxial anaesthesia and general anaesthesia, with multivariate analysis showing no association between general anaesthesia and 5-min Apgar score. Specifically, the authors found no association between general anaesthesia and 5-min Apgar score in their multivariate analysis.^10^ Another study similarly demonstrated no effect of duration of general anaesthesia on neonatal 5-min Apgar scores when adjusted for labour and previous abdominal surgery.^9^ Notably, these studies did not consider the total general anaesthesia dose. Furthermore, an association between hypotension—particularly MAP ≤80 mm Hg—and lower umbilical arterial cord pH has been previously demonstrated in women undergoing planned CD under spinal anaesthesia, a threshold supported by our findings.^19^ This association is likely attributable to low-resistance utero-placental vessels that are reliant on maternal arterial blood pressure to drive maternal blood to the placenta.^34^^,^^35^ Therefore, changes in maternal MAP likely affect fetal well-being and the neonatal 5-min Apgar score.

A little more than half of our cohort required admission to the NICU and the mean length of NICU stay was longer in our study than previously reported (7 days *vs* 4 days)^10^ for neonates of similar gestational age born *via* general anaesthesia. This most likely is explained by the norms at the authors’ institution that require NICU admission of any neonate born at <35 weeks of gestational age, requiring i.v. fluids or requiring any form of respiratory support, including mechanical ventilation *via* intubation, CPAP, or supplemental oxygen. Further, our institution’s NICU will not discharge preterm neonates until their postmenstrual age surpasses 35 weeks’ gestation. The low rates of arterial cord blood pH of <7.10 (3.37%) and base deficit of ≥12 mmol L^−1^ (0%) among the births in this population were also similar to those found among vigorous newborns in the general population.^24^

This study has many notable strengths. Foremost, the unique aspect of this study is that it excluded all emergency and intrapartum CDs where non-reassuring fetal status and the exposure to labour can affect the neonatal Apgar score. There were no missing data for our primary outcome. However, certain limitations should be considered when interpreting these findings. Despite that none of the short-term neonatal outcomes implied any negative effects of general anaesthesia, the long-term neurodevelopmental outcomes could not be studied because of the retrospective nature of the study and limited patient follow-up available in the EMR. Although a MAP threshold of 80 mm Hg appears desirable, this study was not designed to identify a maternal blood pressure threshold, and further prospective research is needed in this area. The EMAC-AUC composite metric necessarily incorporates several approximations—including standardised MAC values, conversion of propofol infusion dose to MAC equivalents, and adjusting the effective MAC in the presence of dexmedetomidine. These assumptions may introduce exposure measurement error. In addition, we could not evaluate all confounding variables such as magnesium use, local anaesthetic exposure, indication for CD, and reasons for general anaesthesia.

Even when anticipated, Caesarean hysterectomy can cause significant maternal psychological stress,^36^^,^^37^ and some, although a minority, patients may prefer general anaesthesia for the entire procedure, including before the birth of the neonate. To minimise prolonged fetal exposure to general anaesthetic agents, preparatory steps (e.g. skin antisepsis and urinary catheter insertion) are often performed on the awake patient, which can also be stressful; such maternal preferences for general anaesthesia should therefore be given due consideration. Although general anaesthesia is generally associated with worse maternal and neonatal outcomes compared with neuraxial anaesthesia,^3^ our findings suggest that when the fetal status is reassuring, urgent delivery after induction of general anaesthesia may not be required.^3^ Instead, careful management of maternal blood pressure may be more critical than minimising general anaesthesia duration to achieving favourable neonatal outcomes. Maintaining MAP >80 mm Hg may reduce the likelihood of low Apgar scores, although prospective studies are needed to confirm this threshold.

## Authors' contributions

Study idea and design: SS, TTK, JAK

Manual data collection: SS, BR, JL, AC

Manuscript preparation: SS, TTK, RS, AC, CP, YY, GM, JAK

Electronic data pull: TTK

Study design: MS, RS, CP

Electronic data collection: MS, NB

Statistical analysis: YY, GM

## Declaration of generative AI and AI-assisted technologies in the writing process

During the preparation of this work, the authors used University of Michigan GPT in order to decrease the word count to adhere to the journal guidelines. After using this tool/service, the authors reviewed and edited the content as needed and takes full responsibility for the content of the publication.

## Funding

Department of Anesthesiology, University of Michigan.

## Declarations of interest

The authors declare that they have no conflicts of interest.
