# Supplementary material for: Association of the dose of maternal general anaesthesia during Caesarean delivery with 5-minute Apgar scores: a retrospective single centre cohort study
Source: BJA Open. 2026 Feb 27;17:100534. doi: 10.1016/j.bjao.2026.100534 (PMC12964278; doi:10.1016/j.bjao.2026.100534)
Supplement: Multimedia component [file mmc1.docx]

Supplemental Table 1: ICD codes used to identify maternal and neonatal comorbidities.

| Placenta accreta spectrum | ICD 9 (PPH and Retained placenta codes) * : PPH: 666.00, 666.02, 666.04  Retained placenta: 667.00, 667.02, 667.04  ICD10: O43.211; O43.212; O43. 221, O43.213; O43.219; O43.231; O43.232; O43.233; O43.23 |
| --- | --- |
| Pre-existing hypertension | ICD 9: 401-405, 642.0-642.2, 642.7 ICD 10: I10-I13, I15, O10, O11 |
| Eclampsia, pre-eclampsia with severe features | ICD 9: 642.5, 642.6. ICD 10: O14, O15 |
| Gestational Hypertension | ICD 9: 642.3 (without pre- eclampsia/eclampsia or pre-existing hypertension) ICD 10: O13, O16 (without pre- eclampsia/eclampsia or pre-existing hypertension) |
| mild pre-eclampsia | ICD 9: 642.4, 642.7 (without severe pre- eclampsia/eclampsia), ICD 10: O11, O14 (without severe pre-eclampsia/eclampsia) |
| Diabetes mellitus | ICD 9: 250., 648.0, 648.8 ICD 10: E10, E11, O24 |
| Diabetes mellitus with complications (Hyperosmolarity, DKA, end organ damage) | ICD 9: 250.1-9, ICD 10: 13.0-13.6, 13.8 |
| Diabetes requiring insulin | ICD10: O24.414, O24.424, O24.434 |
| Transient Tachypnoea of newborn | ICD 9: 770.6, ICD10: P22.1 |
| Maternal care for Congenital anomaly in fetus | ICD 10: O35 |

PPH: Postpartum haemorrhage; DKA: Diabetic ketoacidosis. *Since ICD 9 did not have a code for placenta accreta spectrum we used the codes for postpartum haemorrhage and retained placenta to look for these cases.
