# Supplementary material for: Association of the dose of maternal general anaesthesia during Caesarean delivery with 5-minute Apgar scores: a retrospective single centre cohort study
Source: BJA Open. 2026 Feb 27;17:100534. doi: 10.1016/j.bjao.2026.100534 (PMC12964278; doi:10.1016/j.bjao.2026.100534)
Supplement: Multimedia component [file mmc2.docx]

Supplemental Table 2: Neonatal Outcomes:

| Outcome | **Mean (SD)/N (%)** | **Range** |
| --- | --- | --- |
| 5-minute Apgar score <7 | 36 (35.6%) |  |
| 1-minute Apgar score <7 | 70 (69.3%) |  |
| NICU admission  Very preterm  Moderate preterm  Late preterm  Term | 54 (53.5%)  4(100%)  11(100%)  32 (74.4%)  7(16.3%) |  |
| Neonates requiring intubation  Very preterm  Moderate preterm  Late preterm  Term | 29 (28.7%)  4 (100%)  6 (54.5%)  17 (39.5%)  2 (4.7%) |  |
| CPAP for ≥ 2hrs | 30 (29.7%) |  |
| Oxygen requirement (FiO_2_ ≥0.3) for ≥4 hrs | 7 (6.9%) |  |
| TTN | 17 (16.8%) |  |
| Surfactant treatment | 10 (9.9%) |  |
| Neonatal death within 72 hrs. | 0 |  |
| **Cord blood gas values** |  |  |
| Arterial pH <7.10 | 3 (3.4%) |  |
| Venous pH <7.10 | 1 (1.1%) |  |
| Arterial Base excess ≤ 12 (mmol/L) | 0(0%) |  |
| Venous Base excess ≤ 12 (mmol/L) | 0(0%) |  |
| Duration of NICU stay (days) | 7.36 (18.1) | 0 to 128 |
| Length of intubation (minutes) | 235.59 (824.5) | 0 to 4932 |

SD: Standard deviation; NICU: Neonatal Intensive Care Unit; CPAP: Continuous Positive Airway Pressure; TTN: Transient Tachypnoea of Newborn.
